# Tissue-specific expression of carbohydrate sulfotransferases drives keratan sulfate biosynthesis in the notochord and otic vesicles of *Xenopus* embryos

**DOI:** 10.3389/fcell.2023.957805

**Published:** 2023-03-14

**Authors:** Yuuri Yasuoka

**Affiliations:** ^1^ Laboratory for Comprehensive Genomic Analysis, RIKEN Center for Integrative Medical Sciences, Yokohama, Japan; ^2^ Marine Genomics Unit, Okinawa Institute of Science and Technology Graduate University, Okinawa, Japan

**Keywords:** proteoglycan, chordate bodyplan, gene duplication, evolution, morphogenesis, subfunctionalization

## Abstract

Keratan sulfate (KS) is a glycosaminoglycan that is enriched in vertebrate cornea, cartilage, and brain. During embryonic development, highly sulfated KS (HSKS) is first detected in the developing notochord and then in otic vesicles; therefore, HSKS has been used as a molecular marker of the notochord. However, its biosynthetic pathways and functional roles in organogenesis are little known. Here, I surveyed developmental expression patterns of genes related to HSKS biosynthesis in *Xenopus* embryos. Of these genes, the KS chain-synthesizing glycosyltransferase genes, beta-1,3-N-acetylglucosaminyltransferase (*b3gnt7*) and beta-1,4-galactosyltransferase (*b4galt4*), are strongly expressed in the notochord and otic vesicles, but also in other tissues. In addition, their notochord expression is gradually restricted to the posterior end at the tailbud stage. In contrast, carbohydrate sulfotransferase (Chst) genes, *chst2*, *chst3*, and *chst5.1*, are expressed in both notochord and otic vesicles, whereas *chst1, chst4/5-like*, and *chst7* are confined to otic vesicles. Because the substrate for Chst1 and Chst3 is galactose, while that for others is N-acetylglucosamine, combinatorial, tissue-specific expression patterns of Chst genes should be responsible for tissue-specific HSKS enrichment in embryos. As expected, loss of function of *chst1* led to loss of HSKS in otic vesicles and reduction of their size. Loss of *chst3* and *chst5.1* resulted in HSKS loss in the notochord. These results reveal that Chst genes are critical for HSKS biosynthesis during organogenesis. Being hygroscopic, HSKS forms “water bags” in embryos to physically maintain organ structures. In terms of evolution, in ascidian embryos, *b4galt* and *chst-like* genes are also expressed in the notochord and regulate notochord morphogenesis. Furthermore, I found that a *chst-like* gene is also strongly expressed in the notochord of amphioxus embryos. These conserved expression patterns of Chst genes in the notochord of chordate embryos suggest that Chst is an ancestral component of the chordate notochord.

## Introduction

Glycosaminoglycans (GAGs) are unbranched polysaccharides comprising the extracellular matrix (ECM) in animal tissues. GAGs consist of repeated disaccharide units of acidic sugars and *N*-acetylated amino sugars bearing sulfate groups, resulting in a high negative charge. On the basis of disaccharide composition, GAGs are categorized as heparan sulfate/heparin [D-glucuronic acid (GlcA) or L-iduronic acid (IdoA), and *N*-acetyl-glucosamine (GlcNAc)], chondroitin sulfate [GlcA and N-acetyl-galactosamine (GalNAc)], dermatan sulfate [IdoA and GalNAc], keratan sulfate [D-galactose (Gal) and GlcNAc], and hyaluronic acid [GlcA and GlcNAc] (Prydz and Dalen, 2000). Except for hyaluronic acid, GAGs are initiated on serine or threonine residues of core proteins, forming proteoglycans. A variety of core proteins, GAG types, and modification of GAGs, further increase the complexity of proteoglycans (Iozzo and Schaefer, 2015).

Molecular functions of proteoglycans in embryonic development, tissue differentiation, and disease have been thoroughly analyzed. Heparan sulfate proteoglycans function as scaffolds for cell-cell signal transduction by trapping morphogen ligands such as Bmp, Fgf, Hh, and Wnt (De Pasquale and Pavone, 2019; Mii, 2020). Chondroitin sulfate and dermatan sulfate proteoglycans are enriched in cartilage and brain, forming hydrogels for tissue homeostasis (Roughley and Mort, 2014; Hayes and Melrose, 2021). Keratan sulfate is also enriched in many tissues, such as cornea, central and peripheral nervous systems, and bone and cartilage, functioning in tissue hydration and cell-cell communication (Pomin, 2015; Caterson and Melrose, 2018). Cell type-specific distribution of proteoglycans has been revealed directly with biochemical studies, and indirectly *via* gene expression analysis of core proteins and modification enzymes. Here I focus on biosynthesis of keratan sulfate chains, because little is known about the role of keratan sulfate in early animal development.

**FIGURE 1:**
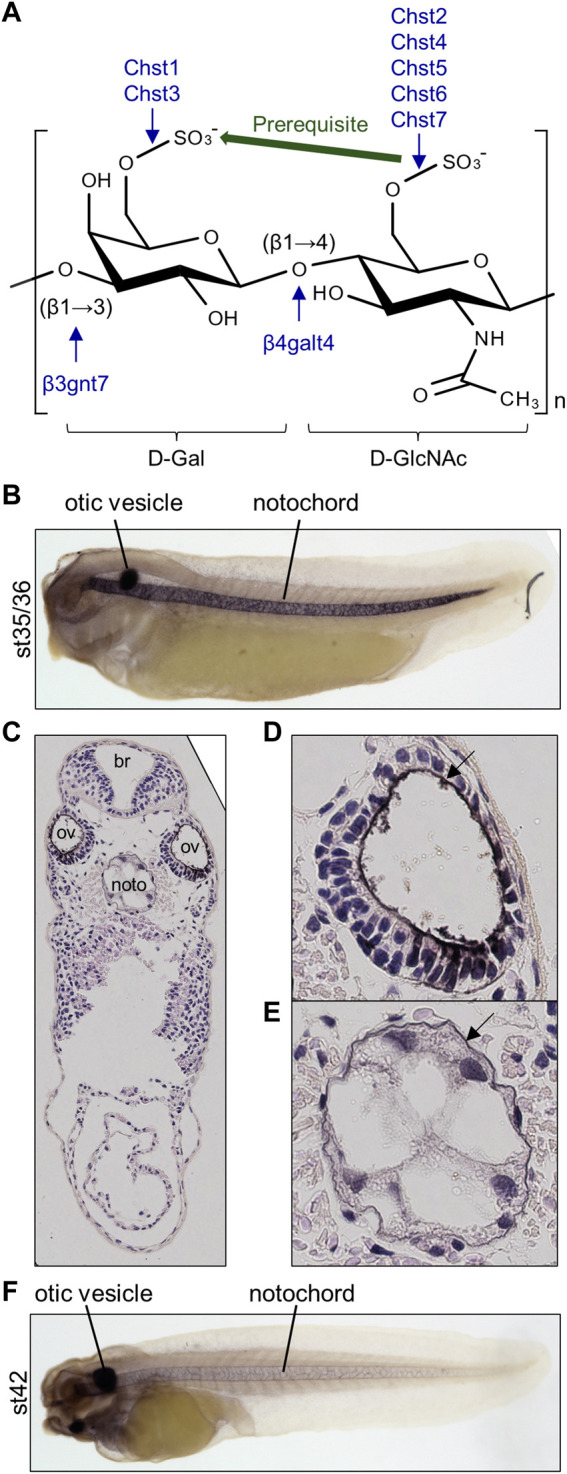
Highly sulfated keratan sulfate (HSKS) is enriched in the notochord and otic vesicles of *Xenopus* embryos. **(A)** Molecular structure of HSKS and enzymes catalyzing its biosynthesis are schematically represented. Sulfation of D-GlcNAc is required for sulfation of D-Gal. **(B–F)** Immunostaining using 5D4 monoclonal antibody demonstrates that HSKS is specifically enriched in the notochord and otic vesicles in *Xenopus tropicalis* tadpoles. In late tadpole stage **(F)**, notochord staining becomes weaker, possibly due to vacuole growth and cell death, which makes the extracellular space smaller. Tadpoles cleared in BB/BA solution (benzyl benzoate: benzyl alcohol = 2:1) are shown in lateral view. **(C–E)** A cross-section of a tadpole (st. 35/36) immunostained with 5D4 antibody revealed that HSKS is enriched inside otic vesicles and outside notochord, as designated by arrows in magnified images of an otic vesicle **(D)** and notochord **(E)**. Br, brain; ov, otic vesicle; noto, notochord.

Keratan sulfate chains are classified as KS-I, II, or III, based on differences in their linkages to core proteins (Caterson and Melrose, 2018). KS-I is attached to asparagine residues of core proteins *via* high mannose *N*-linked glycosylation, whereas KS-II and III are attached to serine/threonine residues of core proteins *via O*-linked glycosylation with *O*-Gal (KS-II) or *O*-mannose (KS-III). Abundance of these types vary between tissues: KS-I, II, and III are enriched in cornea, skeletal tissues, and nervous system, respectively. After initiation processes, keratan sulfate chains are synthesized and elongated by sequential reactions of β-1,3-N-acetyl-glucosaminyltransferase (β3GnT), N-acetylglucosaminyl-6-sulfotransferase (GlcNAc6ST), and β1,4-galactosyl transferase (β4GalT) (Caterson and Melrose, 2018). Then, Gal is sulfated by keratan sulfate galactosyl-6-sulfotransferase (KSGal6ST), activity of which depends heavily on sulfation of GlcNAc (Fukuta et al., 1997; Torii et al., 2000) (Figure 1A). Among seven β3GnT genes and seven β4GalT genes in humans, B3GNT7 and *B4GALT4* encode the most specific enzymes catalyzing keratan sulfate biosynthesis (Seko et al., 2003; Seko and Yamashita, 2004) (Figure 1A). In humans, five GlcNAc6ST genes (*CHST2, CHST4, CHST5, CHST6, CHST7*) and two KSGal6ST genes (*CHST1 and CHST3*) have been identified (Uchimura and Rosen, 2006) (Figure 1A). CHST3 and CHST7 also exhibit chondroitin 6-O sulfation activity (Kitagawa et al., 2000; Yusa et al., 2006).

In *Xenopus* and other vertebrate model systems, highly sulfated keratan sulfate (HSKS) is detected in the developing notochord and otic vesicles of early embryos by specific antibodies such as MZ-15 and 5D4 mouse monoclonal antibodies (Smith and Watt, 1985; Sugimoto et al., 2005) (Figures 1B–F). Therefore, HSKS has been used as a molecular marker of the notochord in developmental biology studies, e.g., detection of axis bifurcation. However, synthetic pathways, molecular functions, and evolutionary origins of HSKS in these tissues have never been investigated. In this study, I assessed these issues using *Xenopus* and amphioxus embryos.

## Materials and methods

### Animal experiments

Adult male and female *Xenopus tropicalis* (Nigerian BH strain) were provided by Hiroshima University Amphibian Research Center through the National BioResource Project (NBRP) of MEXT. All experiments with *X. tropicalis* were approved by the Animal Care and Use Committees at the RIKEN Yokohama Campus and Okinawa Institute of Science and Technology Graduate University.

### Microinjection of *Xenopus* embryos

*Xenopus tropicalis* fertilized eggs were de-jellied and injected with antisense morpholino oligos (MOs). 1 nL of 0.5 mM MOs was injected into the animal pole region of both blastomeres at the two-cell stage (1 pmol per embryo). MOs were purchased from Gene Tools. MO sequences are as follows (antisense start codons are underlined for translation blocking MOs and antisense intron sequences are in small letters for splicing blocking MOs): standard control MO, 5′-CCT​CTT​ACC​TCA​GTT​ACA​ATT​TAT​A-3′; *chst1* MO1, 5′-GCC​TTC​CAA​GAA​CAT​TGCATGGC​TG-3′; *chst1* MO2, 5′-TCT​GCT​GTT​GAC​TCT​GTA​CCA​TAA​G-3′; *chst3* MO1, 5′-tcg​ctg​aca​ttt​ctt​acT​TTG​AGA​T-3′; *chst3* MO2, 5′-TGG​CAA​AGG​GAA​AAT​TCC​AAT​GAC​T-3′; *chst3* MO3, 5′-GAG​AAC​TTC​CTG​TCC​CCC​TTC​ATG​A-3′; *chst5.1* MO1, 5′-TTA​GAG​CCC​GGA​ATC​TGA​CCATGAC-3′; *chst5.1* MO2, 5′-TCC​AGC​TTT​CTG​TTT​TCT​CAG​CCT​C-3′. The *chst1* MO2, *chst3* MO3, and *chst5.1* MO2 were designed as second non-overlapping MOs targeting the 5′ untranslated region (5′UTR). To validate translational or splicing block by MOs, *in vitro* translation or RT-PCR was performed. For RT-PCR, embryos were collected at st. 28. Morphogenetic phenotypes were observed under a stereomicroscope (Zeiss Stemi 305), and otic vesicle sizes were calculated with Labscope v3.1.1 (Zeiss).

### Genome editing of *Xenopus* embryos

To obtain genome-edited embryos, 1 ng of Cas9 protein (IDT, Alt-R^®^ S.p. HiFi Cas9 Nuclease V3) and 200 ng of sgRNA were co-injected into the animal pole region of *X. tropicalis* embryos at the 1-cell stage. sgRNA was synthesized from PCR-assembled template DNA by *in vitro* transcription using a MEGAshortscript™ T7 Transcription Kit (Thermo Fisher Scientific, AM1354) as described (Nakayama et al., 2013; Sakane et al., 2017; Blitz and Nakayama, 2022). For PCR assembly of the template DNA, the 5′ oligonucleotide (5′-TAATACGACTCACTATAGG(N)_18_GTTTTAGAGCTAGAAATAGCAAG-3′, (N)_18_ corresponding to the target sequence in each gene of interest) and 3′ oligonucleotide (5′-AAA​AGC​ACC​GAC​TCG​GTG​CCA​CTT​TTT​CAA​GTT​GAT​AAC​GGA​CTA​GCC​TTA​TTT​TAA​CTT​GCT​ATT​TCT​AGC​TCT​AAA​AC-3′) were used. Genome editing efficiency of each embryo was examined at the early tadpole stage using a DNeasy Blood & Tissue Kit (QIAGEN) for DNA extraction and an Alt-R^®^ Genome Editing Detection Kit (IDT) for T7 endonuclease I (T7E1) assays. sgRNA for the *tyrosinase* gene [(N)_18_ is AAC​TGG​CCC​CTG​CAA​ACA] was used as a control. sgRNA sequences were designed using CRISPRdirect (Naito et al., 2015), checking their specificity in the *X. tropicalis* genome v10 (see Supplementary Tables S1–S3). *In vitro* cleavage of target DNA with the Cas9-sgRNA complex was performed as described in the IDT protocol with a small modification, in which CutSmart buffer (NEB) was used to prepare the ribonucleoprotein complex and to digest DNA.

### Whole mount *in situ* hybridization

Coding sequences of *X. tropicalis* genes and *Branchiostoma floridae* genes were PCR-amplified from cDNA pools of embryos and cloned into pCSf107 mT vectors (Mii and Taira, 2009) using an In-Fusion HD Cloning kit (Takara). Whole-mount *in situ* hybridization of *Xenopus* and amphioxus embryos was performed as previously described (Harland, 1991; Yu and Holland, 2009; Yasuoka et al., 2014), using digoxigenin-labelled anti-sense probes, which were transcribed from linearized plasmids. For *Xenopus* embryos, automated hybridization experiments were performed with InsituPro VSi (Intavis). Stained *Xenopus* embryos were bleached and observed under a stereomicroscope (Leica M205 FA). Stained amphioxus embryos were observed under a fluorescence microscope (Keyence BZ-X810).

### Whole mount immunostaining

Whole-mount immunostaining was performed as described (Suga et al., 2006; Yasuoka et al., 2009; Yasuoka et al., 2014) with modifications for fluorescent imaging. Briefly, embryos were bleached before staining. 5D4 antibody (mouse monoclonal IgG) was used as the primary antibody (Cosmo Bio, PRPG-BC-M01, 1/100 diluted). HRP-conjugated anti mouse IgG (Promega, 1/500 diluted) or AlexaFluor488-conjugated anti-mouse IgG (Thermo Fisher Scientific, A-11001, 1/200 diluted) was used as the secondary antibody. For HRP staining, a Peroxidase Stain DAB Kit and Metal Enhancer for DAB Stain (Nacalai Tesque) were used. Automated immunolabelling experiments were performed with InsituPro VSi (Intavis). Some DAB-stained embryos were subjected to cross-sections stained with hematoxylin. Stained embryos were observed under a fluorescence microscope (Leica M205 FA or Keyence BZ-X810).

### Phylogenetic analysis

To identify putative deuterostome Chst genes, protein-coding DNA sequences of *X. tropicalis* Chst1, Chst2, and Chst3 were submitted as queries to ORTHOSCOPE (v1.5.2), a species tree-based ortholog identification tool (Inoue and Satoh, 2019), with the following settings: analysis group, Deuterostomia; E-value threshold for reported sequences, 1e^−5^; number of hits to report per genome, 20; aligned site rate threshold within unambiguously aligned sites, 0.55; data set, DNA (Exclude third); rearrangement BS (bootstrap) value threshold, 60%. Using amino acid sequences reported by ORTHOSCOPE, an ML tree was constructed as described (Yasuoka et al., 2020).

### Transcriptomic analysis

To examine expression profiles of *chst16* during *Xenopus* development, I re-analyzed public time-course RNA-seq data (Owens et al., 2016). Fastq data of SRR1795535-1795624 (Clutch A polyA RNA-seq, 0-66 hpf) were mapped to the *X. tropicalis* genome v10.0 with NCBI gene models using STAR v2.7.1a (Dobin et al., 2013), and transcripts per embryo were calculated using RSEM v1.2.28 (Li and Dewey, 2011).

## Results

### HSKS is enriched inside otic vesicles and outside the notochord

To determine the suborgan distribution of HSKS, I observed cross-sections of *X. tropicalis* embryos immunostained with 5D4 antibody at the early tadpole stage (Figures 1C–E). The result shows that HSKS is enriched in the ECM layer lining the lumen of otic vesicles (Figure 1D). On the other hand, HSKS is enriched in the ECM layer outside the notochord, which is called the notochordal sheath, but not in the vacuoles (Figure 1E). At later stages, HSKS is still abundant in otic vesicles, but decreases in the notochord, in which vacuoles grow larger (Figure 1F). These observations indicate that otic vesicles and notochord utilize HSKS for tissue hydration in different ways. Therefore, the lumen of otic vesicles must have evolved independently of the notochordal vacuole in vertebrates. Genetic mechanisms underlying these differences are further analyzed below.

### HSKS synthetic genes are temporally syn-expressed during *Xenopus* development

To examine fundamental roles of HSKS in early vertebrate embryos, I focused on expression profiles of the synthetic pathway genes, *b3gnt7*, *b4galt4*, *chst1*, *chst2*, *chst3*, *chst4/5-like*, *chst5.1*, and *chst7* in *X. tropicalis* embryos. Names of carbohydrate sulfotransferase (chst) genes are in accordance with a recent comprehensive phylogenetic study of the carbohydrate 6-O sulfotransferase gene family (Daza and Haitina, 2020), in which *chst4*, *chst5*, and *chst6* are renamed on the basis of their phylogenetic relationships (see Table 1; Supplementary Figures S1–S4 for details). Notably, *chst4* and *chst6* are lineage-specific paralogs of the *chst4/5/6* gene in tetrapods and primates, possibly produced by local gene duplication. Another *chst4/5-like* gene is present in amphibians and some lepidosaurs, but its origin is uncertain. In frogs, *chst5* is further duplicated into *chst5.1 and chst5.2.* Daza and Haitina (2020) reported that *chst4* was lost and that *chst5.1* and *chst5.2* are present in the *X. tropicalis* genome, but the current genome assembly of *X. tropicalis* (v10) revealed the presence of *chst4* and *chst5.1* and the absence of *chst5.2*, with conserved microsyntenies around these genes (Supplementary Figures S1–S3). Evidently, the previous study overlooked *X. tropicalis chst4*, which is present in the genome assembly used (v9.1) (see Supplementary Figure S1). The presence of *chst4* is plausible, since all other tetrapod genomes retain the gene, suggesting that it serves an indispensable role. The absence of *chst5.2* in the current genome assembly is enigmatic, but this gene may be dying or may be undergoing neofunctionalization, given its accelerated evolutionary rate compared to *chst5.1* (Daza and Haitina, 2020).

**TABLE 1:** Modified gene names from the database (Xenbase and NCBI Entrez Gene).

Names in this study	*X. tropicalis*	*X. laevis L*	*X. laevis S*
*chst4*	*chst5*	*chst5.L*	*chst5.S*
*chst4/5-like*	*chst4*	a	*chst4.S*
*chst5.1*	*chst6*	*chst6.L*	*chst6.S*
*chst5.2*	a	*LOC108714029*	*LOC108715076*
*chst16*	*LOC100485856*	*LOC108710801*	a

^a^ , gene losses.

**FIGURE 2:**
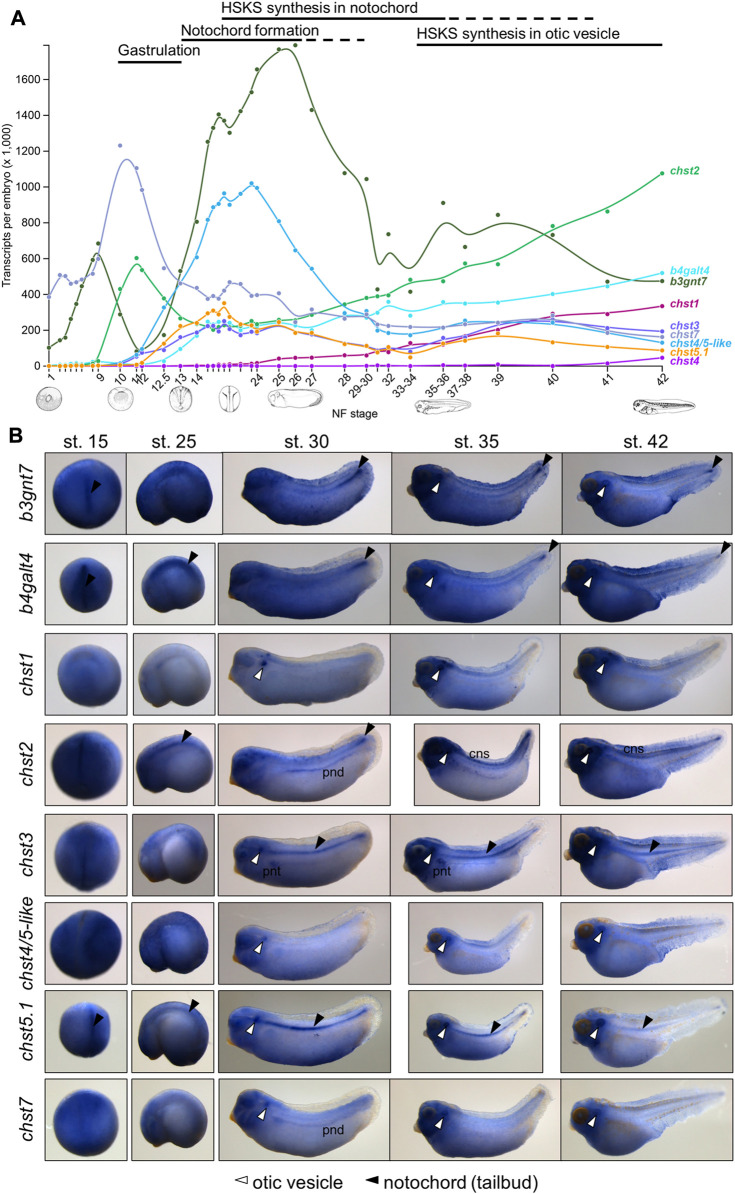
Spatio-temporal expression patterns of HSKS catalytic genes are linked to HSKS synthesis during *Xenopus* development **(A)** Expression levels of HSKS biosynthetic genes along the developmental time-course of *Xenopus* embryos are visualized in Xenbase (https://www.xenbase.org/) using an available RNA-seq dataset (Owens et al., 2016). Most genes showed elevation of expression levels corresponding to enrichment of HSKS in the notochord and otic vesicles. **(B)** Spatial expression patterns of *Xenopus* HSKS biosynthetic genes are represented with whole mount *in situ* hybridization from neurula to tadpole stages (st. 15–42). To detect their expression in the notochord, some embryos were overstained, resulting in higher background, especially in the head region of tadpoles. Therefore, it is difficult to distinguish precise expression domains of genes strongly expressed in the brain such as *chst1*, *chst2*, and *chst3*. Arrowhead, notochord; open arrowhead, otic vesicles; cns, central nervous system; pnd, pronephric duct; pnt, pronephric tubules.

Using publicly available time-course transcriptomic data during early embryogenesis of *X. tropicalis* (Owens et al., 2016), I first examined temporal expression patterns of HSKS synthetic pathway genes in *Xenopus* development (Figure 2A). The data showed that *b3gnt7* and *chst7* are maternally expressed, whereas others are zygotically expressed. Among zygotically expressed genes, *chst2* expression initiates at the early gastrula stage (stage 10), earlier than others, whereas *chst1* expression gradually appears from the pharyngula stage (stage 25). *chst4* is almost silent during early embryogenesis, but is weakly expressed at the tadpole stage (stage 42). Remarkably, HSKS synthetic pathway genes, except *chst1* and *chst4*, exhibit temporal synexpression patterns, corresponding to HSKS synthesis in the notochord and otic vesicles (Figures 1B–F). Because it was reported that temporal synexpression can be used to predict common gene functions in embryonic tissues (Owens et al., 2016), temporal synexpression of HSKS synthetic pathway genes suggests their coordinated functions during development*.*

### Chst genes are expressed in the notochord and otic vesicles

Next, I examined spatial expression patterns of those enzymes by whole-mount *in situ* hybridization (Figure 2B). These data showed that each gene has distinct tissue-specific expression patterns. Among them, glycosyltransferases (*b3gnt7* and *b4galt4*) are relatively ubiquitously expressed with strong expression in the notochord from neurula (st. 15) to pharyngula (st. 25) stages and in the tailbud region and otic vesicles at tadpole stages (st. 30–42).

Chst genes showed more restricted expression patterns (Figure 2B). *chst1* manifests highly specific expression in otic vesicles and a small region of hindbrain. Similarly, *chst4/5-like* and *chst7* are specifically expressed in otic vesicles. On the other hand, *chst3* and *chst5.1* are specifically expressed in the notochord and otic vesicles. *chst2* presents a dynamic, complicated pattern. Its expression occurs in the notochord from neurula to pharyngula, but is then restricted to the tailbud region at early tadpole stage (st. 30). Finally, it is detected in otic vesicles and the central nervous system (st. 35–42). Notably, *chst2* and *chst7* are also expressed in the pronephric duct and *chst3* is present in pronephric tubules. Similar expression patterns of *chst2* and *chst7* in tadpole stages (st. 30–42) may reflect their evolutionary relationship as “ohnologs”, paralogs generated by whole-genome duplication in vertebrates (Daza and Haitina, 2020). Consistent with the decreasing amount of HSKS in the notochord at late tadpole stage (Figures 1B–F), expression levels of HSKS synthetic genes are reduced.

These expression patterns of Chst genes suggest that tissue-specific expression of Chst genes is responsible for HSKS biosynthesis in the notochord and otic vesicles. The absence of HSKS in the pronephros is plausible because GlcNAc6ST (*chst2/7*) and KSGal6ST (*chst3*) are not co-expressed there, suggesting that Chst2/7 and Chst3 catalyze sulfation of different molecules in the pronephric system. In addition, *chst1*, *chst2*, and *chst3* are also strongly expressed in the central nervous system, mainly brain, but HSKS is hardly detected in brains of tadpoles (Figures 1B–F), suggesting that those enzymes work in different parts of the brain and/or catalyze different substrates. Here I focused on the notochord and otic vesicles in early-stage embryos, but more detailed expression analysis of these enzymes in the pronephros and brain will reveal the biosynthetic pathway of HSKS in those tissues.

A recent comprehensive phylogenetic analysis of the carbohydrate 6-O sulfotransferase gene family revealed that frogs retain the *chst16* gene, an ohnolog of *chst1*, which was lost in amniotes (Daza and Haitina, 2020). In the current *X. tropicalis* genome assembly (v10), *chst16* is located on chromosome 3 and annotated as *LOC100485856* (Tale 1 and Supplementary Figure S5A). In *Xenopus laevis*, *chst16.S* is possibly lost and *chst16.L* is annotated as *LOC108710801* (Table 1). Otic vesicle expression of neighboring genes, *tmem263.L* and *cry1.L,* implies that *chst16* is also expressed in otic vesicles under co-regulation in the same topologically associated domain (TAD) with *tmem263* and *cry1* (Supplementary Figure S5). However, in contrast to its ohnologs (*chst1* and *chst3*), mRNA expression of *chst16* is hardly detected in the time-course transcriptome data of *X. tropicalis* embryos (Supplementary Figure S6), suggesting that *chst16* scarcely contribute to HSKS biosynthesis in early embryos.

### Chst1, Chst3, and Chst5.1 have indispensable roles in HSKS biosynthesis during *Xenopus* development

**FIGURE 3:**
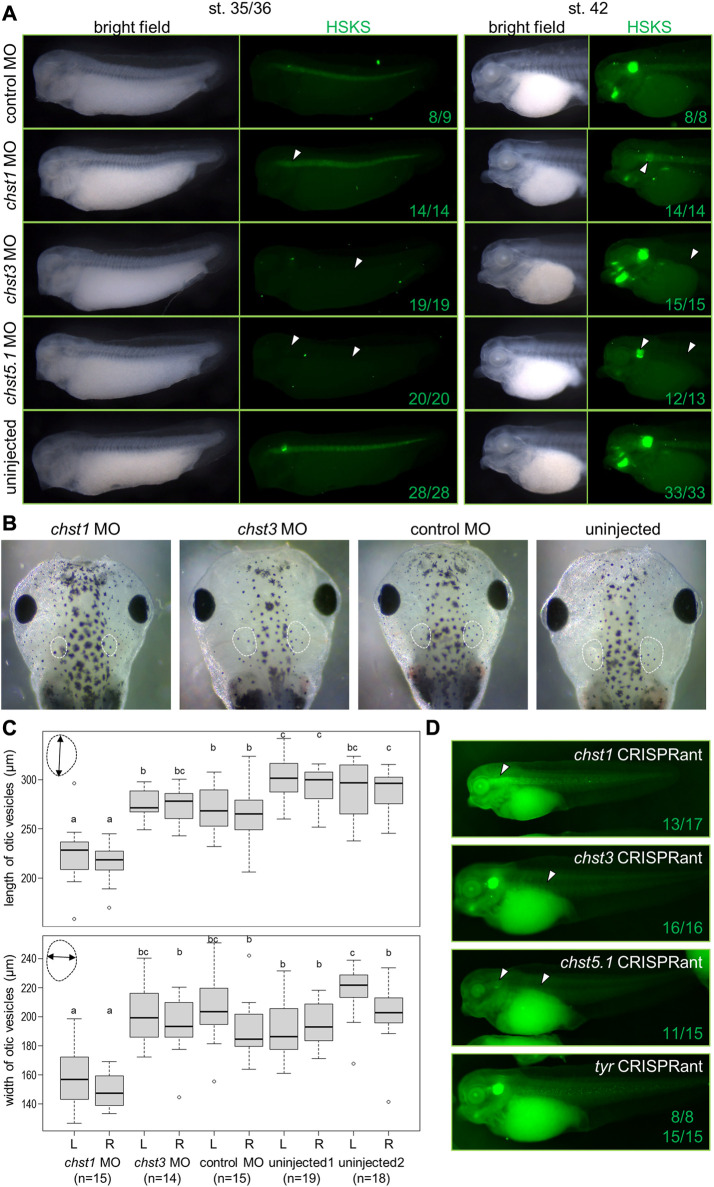
Loss of function analysis revealed indispensable functions of Chst1, Chst3, and Chst5.1 for HSKS synthesis in *Xenopus* embryos. **(A)** Fluorescent immunostaining of HSKS of morphants at st. 35/36 and 42. Open arrowheads indicate lost or reduced enrichment of HSKS. See the text for a detailed explanation of phenotypes. **(B)** Smaller otic vesicles were observed in *chst1* morphants at st. 45. White dashed lines indicate outlines of otic vesicles **(C)** Quantification of otic vesicle phenotypes. Box plots indicate length and width of otic vesicles on the left (L) or right (R) side in each sample. Each value (length of left vesicle, length of right vesicle, width of left vesicle, and width of right vesicle) was statistically analyzed with one-way ANOVA (*p* < 6.2 E^−13^, 9.9 E^−11^, 6.4 E^−16^, and 9.6 E^−13^, respectively), followed by Tukey’s honestly significant difference test with 95% confidence level (indicated with a, b, and c). The result demonstrated that otic vesicles of *chst1* morphants are significantly smaller than those of *chst3* morphants, control morphants, and uninjected controls **(D)** Genome editing experiments using the CRISPR-Cas9 system further demonstrated that *chst1*, *chst3*, and *chst5.1* serve indispensable functions in HSKS in otic vesicles, notochord, and both, respectively (see Supplementary Figures S9–11 for more details). Numbers of embryos with observed phenotypes are indicated.

**FIGURE 4:**
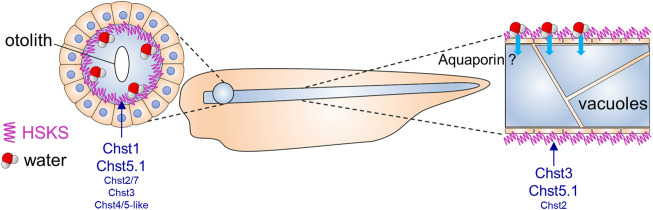
Putative roles of HSKS in early *Xenopus* development.The contribution of HSKS to tissue hydration in *Xenopus* tadpoles is schematically represented. Accumulated HSKS in extra cellular matrix retains water, which may help otic vesicles and notochord vacuoles to swell. HSKS is enriched inside otic vesicles (Figure 1D), but outside of the notochord (Figure 1E), implying that aquaporin transports water from the extracellular space to intracellular vacuoles. Our results demonstrate that tissue-specifically expressed *chst* genes drive HSKS biosynthesis for normal development.

To examine Chst roles in HSKS formation, I performed loss of function experiments by microinjection of antisense morpholino oligos using *X. tropicalis* embryos (Figure 3A, see Supplementary Figures S7, S8 for validation of knock-down efficiency and specificity of morpholinos). Consistent with their expression domains, *chst1* morphants exhibit loss of HSKS in otic vesicles, whereas *chst3* and *chst5.1* morphants do not produce HSKS in the notochord. The presence of HSKS in otic vesicles of *chst3* and *chst5.1* morphants indicates that other Chst genes compensate for their loss to synthesize HSKS in otic vesicles. Compared with *chst3* morphants, *chst5.1* morphants exhibit a more severe phenotype with reduced HSKS in otic vesicles. These results suggest that *chst5.1* is the main contributor of GlcNAc6ST activity in both notochord and otic vesicles, and that *chst1* and *chst3* contribute to KSGal6ST activity in otic vesicles and notochord, respectively (Figure 4).

To validate specificities of morpholinos upon each gene function, second non-overlapping morpholinos were injected to *X. tropicalis* embryos. Because coding sequences of *chst1* and *chst5.1* are in single exons, splicing blocking morpholinos are unavailable for them. Therefore, *chst1* MO2 and *chst5.1* MO2 were designed to bind the 5′UTR to block translation (Supplementary Figure S7). Embryos injected with *chst1* MO2 showed loss of HSKS in otic vesicles, further ensuring the HSKS synthetic function of Chst1 in otic vesicles. On the other hand, embryos injected with *chst5.1* MO2 died during gastrulation, possibly due to its cytotoxicity. For *chst3*, two translation blocking morpholinos were examined (Supplementary Figure S8). Although both morpholinos were cytotoxic to some extent, *chst3* MO3 is relatively safer than *chst3* MO2, and embryos injected with *chst3* MO3 exhibited reduction of HSKS in the notochord, supporting the hypothesis that Chst3 synthesizes HSKS in the notochord.

To investigate the impact of reduced HSKS on morphogenesis, otic vesicle sizes were compared between morphants at later tadpole stage (Figure 3B). Quantitative data regarding otic vesicle size indicate that otic vesicles of *chst1* morphants are significantly smaller than other morphants and uninjected controls (Figure 3C). Together with the reduction of HSKS levels specifically in otic vesicles by *chst1* knockdown (Figure 3A), HSKS effects on otic vesicle formation are assumed to retain water for lumen growth of otic vesicles. Despite the smaller size of otic vesicles, *chst1* morphants formed otoliths normally, suggesting that HSKS does not affect the composition of the liquid inside otic vesicles.

Furthermore, I have also discovered a bent axis phenotype in *chst5.1* morphants (Supplementary Figure S7F). This phenotype became evident gradually from early to late tadpole stages. Loss of HSKS in notochord of *chst5.1* morphants may have impaired water retention and durability of the notochord, resulting in a bent axis. On the other hand, *chst3* morphants did not show similar phenotypes, possibly due to substrate specificity. Monosulfated forms of keratan sulfate should remain in *chst3* morphants and may be sufficient to permit the notochord to support the embryonic axis. In fact, keratan sulfates in human cornea comprise ∼4% unsulfated, 42% monosulfated, and 54% disulfated disaccharides (Plaas et al., 2001), suggesting that the monosulfated form is functional to some extent. More detailed comparison of notochord morphology, e.g., vacuole shape and size, will reveal molecular functions of *chst3*, *chst5.1* and others in notochord development.

To further validate the functions of *chst1*, *chst3*, and *chst5.1* in *X. tropicalis* embryos, I also performed genome editing experiments using the CRISPR-Cas9 system (Supplementary Figures S9–11). I designed two to four sgRNAs for each gene and their activity was confirmed by *in vitro* cleavage of target DNA. Then, preincubated Cas9-sgRNA complex was microinjected into fertilized eggs. Although some sgRNAs hardly exerted genome editing activity in embryos, I found several reliable sgRNAs for each gene. Consistent with morphant phenotypes, most of *chst1*, *chst3*, and *chst5.1* CRISPRants exhibited loss of HSKS in the otic vesicle, notochord, and both, respectively (Figure 3D). Furthermore, otic vesicles of *chst1* CRISPRants were smaller than those of control embryos, recapitulating the *chst1* morphant phenotype (Figure 3C; Supplementary Figure S9G). Although bent axis phenotypes were not observed in F0 embryos of *chst5.1* CRISPRants, the *chst5.1* function in axial morphogenesis could be confirmed by examining their F1 or F2 embryos.

### A *chst* gene is strongly expressed in amphioxus notochord

In tunicate (*Ciona intestinalis*) embryos, a glycosyltransferase gene (*Ci-b4galt*) and two carbohydrate 6-O sulfotransferase genes (*Ci-C6ST-like1* and *Ci-C6ST-like7*) also exhibit restricted expression in the notochord (Katikala et al., 2013; Nakamura et al., 2014), suggesting that HSKS biosynthetic genes have participated in notochord formation since chordates arose. In fact, abnormal morphogenesis of the notochord was caused by knockdown of *Ci-C6ST-like1* and *Ci-C6ST-like7* (Nakamura et al., 2014). Because tunicate *chst* genes are quite distant from vertebrate genes (Daza and Haitina, 2020), substrates of Ci-C6ST-like1 and Ci-C6ST-like7 have not yet been determined. Enzymatic activity of Chst, such as GlcNAc6ST and KSGal6ST, may have evolved independently in each lineage.

**FIGURE 5:**
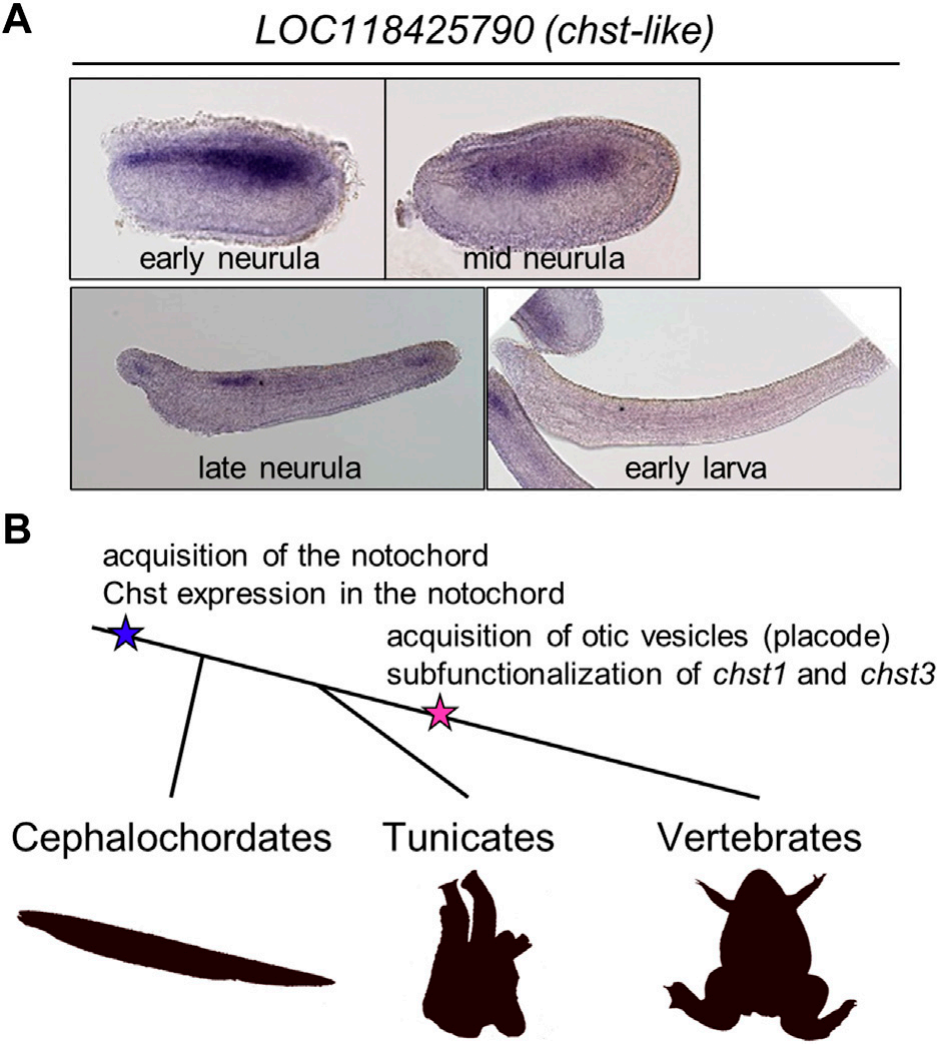
A *chst* gene is expressed in amphioxus notochord, suggesting evolutionary conservation of HSKS functions in chordates. **(A)** Whole-mount *in situ* hybridization using amphioxus embryos showed that expression of a putative Chst gene *LOC118425790* is restricted to the notochord and a part of the neural tube. **(B)** An evolutionary scenario related to Chst genes in chordates. In this scenario, Chst expression is an ancestral feature of the notochord, and in association with the acquisition of otic vesicles, *chst1* and *chst3* (and others, possibly) are subfunctionalized to catalyze biosynthesis of HSKS in various tissues.

To address the evolutionary origin of Chst gene expression in the notochord more deeply, I investigated expression patterns of Chst genes in amphioxus embryos (*B. floridae*). First I searched amphioxus Chst genes using ORTHOSCOPE (Inoue and Satoh, 2019). ORTHOSCOPE extracted 15 putative Chst genes from *B. floridae* gene models (assembly annotation: Bfl_VNyyK) (Supplementary Figure S12). Among them, six genes were annotated as “*carbohydrate sulfotransferase 1-like*,” seven were “*carbohydrate sulfotransferase 3-like*,” one was “*carbohydrate sulfotransferase 5-like*,” and one was “*dermatan-sulfate epimerase-like*”, but these gene names do not represent orthologous relationships to corresponding vertebrate genes, as shown by the phylogenetic analysis (Supplementary Figures S12, 13). To validate their expression patterns, four genes were successfully cloned by RT-PCR using a cDNA pool of *B. floridae* embryos (mid-neurula to mid-larva). Finally, I found that a Chst gene, *LOC118425790*, is strongly expressed in developing notochord of amphioxus neurulae to larvae (Figure 5A). This result suggests that notochord-specific expression of Chst genes is an ancestral feature of chordates (Figure 5B).

## Discussion

In this study, I have demonstrated that HSKS biosynthetic genes are temporally and spatially syn-expressed for HSKS formation in the notochord and otic vesicles of *Xenopus* embryos (Figures 1, 2). Loss of function analysis revealed indispensable roles of *chst1*, *chst3*, *and chst5.1* in HSKS synthesis, in accordance with their expression domains (Figures 2B, 3A). The small otic vesicle phenotype of *chst1* morphants and CRISPRants demonstrated that HSKS is important for tissue morphogenesis (Figure 3C; Supplementary Figures S9G). These functions remain to be examined in more detail using other genetic tools such as genome editing. Although HSKS abundance has long been recognized in the notochord and otic vesicles, this is the first molecular demonstration of tissue-specific expression and functions of HSKS biosynthetic enzymes in vertebrate embryos. Various genetic programs underlying HSKS biosynthesis in the notochord and otic vesicles are feasible by virtue of their different developmental and evolutionary origins. The notochord develops from dorsal midline mesoderm and originates from a chordate ancestor, but otic vesicles develop from otic placode and originate from a vertebrate ancestor (Figure 5B). Furthermore, I have also shown that a Chst gene is strongly expressed in the amphioxus notochord (Figure 5A), suggesting that HSKS in the notochord is an ancestral feature of chordates and that the biosynthetic program was coopted to otic vesicles in vertebrates (Figure 5B).

### Chst genes may have been subfunctionalized in vertebrates and other deuterostomes

Co-option of the HSKS biosynthetic pathway to otic vesicles may have resulted from subfunctionalization of Chst ohnologs. For example, *chst1* and *chst3* share KSGal6ST activity, but their expression domains are very different (Figures 1A, 2B). Similarly, *chst2*, *chst4/5-like*, *chst5.1*, and *chst7* appear to be subfunctionalized with the same catalytic activity (GlcNAc6ST) (Figures 1A, 2B). In zebrafish, *chst1* is specifically expressed in otic vesicles at the 14–19 somite stage (Thisse and Thisse, 2004). *chst3a* shows relatively ubiquitous expression with strong expression in somites and notochord at the five- and 15-somite stages, whereas *chst3b* displays only very weak expression (Habicher et al., 2015). *chst5* (annotated as *chst6* in NCBI and ZFIN databases) appears to be expressed in notochord and otic vesicles at somitogenesis stages. *chst7* is strongly expressed in the notochord and tailbud at the five- and 15- somite stages and also in otic vesicles at the pharyngula stage (24 hpf) (Habicher et al., 2015). Taken together with my *Xenopus* data (Figure 2B), regulatory networks to control *chst1* and *chst5* expression may have been conserved in vertebrates, whereas those for *chst3* and *chst7* vary among amphibians and teleosts. Therefore, subfunctionalization of *chst* genes may have occurred multiple times independently, as long as HSKS are normally synthesized.

Although a previous study proposed that the vertebrate ancestor possessed an ancestral gene set comprising *chst1/16*, *chst3*, *chst2/7*, and *chst4/5* before two rounds of whole-genome duplication (2R-WGD) (Daza and Haitina, 2020), I assume a more simplified ancestral gene set with one KSGal6ST gene (*chst1/3/16*) and one GlcNAc6ST gene (*chst2/4/5/7*). In this scenario, after the first WGD, *chst1/16* and *chst3* may have been subfunctionalized for development of otic vesicles and notochord (Figures 4, 5B). Similarly, subfunctionalization of *chst2/7* and *chst4/5* may have occurred in an early stage of vertebrate evolution. Subtle differences of amino acid sequences surrounding adenosine 3′-phosphate 5′-phosphosulfate binding motifs (Ong et al., 1999) in Chst proteins may have been associated with differentiation of substrates and their subfunctions (Supplementary Figure S14). More comprehensive studies on genomic synteny, expression profiles and substrate specificity should provide answers to these scenarios.

Because there are no clear orthologs of *chst1/3/16* and *chst2/4/5/7* in invertebrate deuterostome genomes (Supplementary Figures S12, 13), all *chst* genes for HSKS synthesis in vertebrates are probably derived from lineage-specific gene/genome duplication events. Notably, deuterostome *chst* genes tend to be duplicated in each lineage, as exemplified by 24, 11, 15, and 7 putative *chst* genes in acorn worms (*Saccoglossus kowalevskii*), starfish (*Acanthaster planci*), amphioxus (*B. floridae*), and ascidians (*C. intestinalis*), respectively (Supplementary Figures S12, 13). This character may have allowed GlcNAc6ST, KSGal6ST, and other Chst genes to evolve independently, and to increase complexity of biosynthetic reactions of GAG chains.

### HSKS roles in development remain to be discovered

Here I address the biosynthetic pathway of HSKS, but core proteins for HSKS in notochord and otic vesicles are still unknown. It is possible that different core proteins contribute scaffolds of HSKS in the notochord and otic vesicles, as demonstrated for Chst genes in this study. In addition, not only highly sulfated forms of keratan sulfate, but also less sulfated forms should have some functions in embryos. Axis malformation in *chst5.1* morphants, but not in *chst3* morphants, suggests significant roles of less sulfated keratan sulfate (Figure 3D). Expression of *chst2* and *chst7* in the pronephric duct (Figure 2B) suggest enrichment of keratan sulfate chains (without sulfation of galactose) in that tissue. Together with *chst3* expression in pronephric tubules, molecular functions of keratan sulfate and other sulfated GAGs in the pronephric system remain to be discovered. In terms of water metabolism, there may be similarities between the function of HSKS in the pronephric system and that in otic vesicles and notochord.

The function of HSKS in tissue morphology remains largely unknown, although some of it is revealed by this study. In zebrafish embryos, HSKS is not enriched in vacuoles of notochord cells, but in extracellular spaces (Ellis et al., 2013), which is consistent with our result in *Xenopus* (Figure 1E). Therefore, HSKS does not contribute to vacuolation of the notochord by serving as an osmolyte. Instead, I surmise that HSKS functions in tissue hydration to maintain turgor pressure of the notochord to support its rod-like structure (Figure 4). It has been shown that the highly sulfated form of keratan sulfate is enriched in cornea and cartilage and functions in tissue hydration (Roughley and Mort, 2014; Caterson and Melrose, 2018; Puri et al., 2020). Therefore, HSKS may also serve to keep water in the notochord and otic vesicles to maintain their morphology. That is, the notochord is a vacuolated support presenting a rod-like structure, and otic vesicles are spherical hollow organs forming otoliths inside. They are like water bags in embryos. In the case of notochord, aquaporin may be involved in transport of water absorbed by HSKS, although such functions of aquaporin genes in early notochord development have never been examined. As I proposed previously (Yasuoka, 2020), it is important to balance turgor pressure and sheath strength during notochord morphogenesis. HSKS may contribute to both by tissue hydration and extracellular matrix formation. In addition, HSKS may also have crucial roles in establishment of the biomineralization environment in the otic vesicle.

In *Ciona*, *chst* genes (*Ci-C6ST-like1* and *Ci-C6ST-like7*) are required for notochord morphogenesis (Nakamura et al., 2014), but localization of HSKS has not been examined. Since *Ciona* notochord forms multicellular hollow tubes instead of becoming vacuolated (Dong et al., 2009), it would be valuable to investigate whether HSKS accumulates in the lumen, which is extracellular in *Ciona* notochord. Similarly, localization of HSKS in amphioxus embryos has not been analyzed. Together with functional assays of a *chst* gene expressed in the notochord (Figure 5A), further analysis of amphioxus embryos is needed to understand fundamental roles of HSKS in chordates.

Another role of HSKS could be signal transduction, as with other GAGs. Interaction between HSKS and signaling molecules such as Shh and Fgf2 has been reported (Weyers et al., 2013), and Chst2 KO mice were impaired in neural tube patterning by Shh signaling (Hashimoto et al., 2016). Compared to chondroitin sulfate and hyaluronic acid, keratan sulfate interacts with a larger number of neuroregulatory proteins such as Slit, Ephrin, and Semaphorin (Conrad et al., 2010; Melrose, 2019). Therefore, HSKS may modulate signal transduction by binding to ligands and receptors for inductive signals from notochord.

### *CHST3* is a susceptibility gene for lumbar disc degeneration

A genome-wide association study revealed that lumbar disc degeneration is associated with a variant (rs4148941) in the 3′UTR of *CHST3* (Song et al., 2013). This disease-susceptibility allele enhances binding of a microRNA (miR-513a-5p) to the 3′UTR and reduces mRNA expression levels of *CHST3* in annulus fibrosus, cartilage end-plate, and nucleus pulposus. Because the nucleus pulpo sus of intervertebral discs is a notochord remnant tissue, my finding of *chst3* functions for HSKS synthesis in the notochord may be relevant to human diseases. That is, evolutionary and developmental remnant cells may be responsible for human back pain.

## Conclusion

HSKS proteoglycans are synthesized through sequential reactions catalyzed by β3GnT, GlcNAc6ST, β4GalT, and KSGal6ST and are enriched in the notochord and otic vesicles of early vertebrate embryos. Some carbohydrate sulfotransferases exert GlcNAc6ST or KSGal6ST activity and their expression is restricted to those tissues. Remarkably, two KSGal6ST genes, *chst1* and *chst3*, are differentially expressed and required for HSKS synthesis in otic vesicles and notochord in *Xenopus* embryos, suggesting subfunctionalization after gene/genome duplication. Notochordal expression of an amphioxus Chst-like gene further suggests that Chst contributed to chordate notochord development. Further studies on molecular functions of HSKS in development, and evolutionary comparisons between vertebrates and invertebrates should provide great insight into the origin of the notochord and contributions of glycobiology to human diseases.

## Data Availability

The original contributions presented in the study are included in the article/Supplementary Materials, further inquiries can be directed to the corresponding author.
