# Supplementary material for: Tissue-specific expression of carbohydrate sulfotransferases drives keratan sulfate biosynthesis in the notochord and otic vesicles of Xenopus embryos
Source: Front Cell Dev Biol. 2023 Mar 14;11:957805. doi: 10.3389/fcell.2023.957805 (PMC10043435; doi:10.3389/fcell.2023.957805)
Supplement: Supplementary file 18 [file DataSheet1.docx]

## Figure S1. Genome browser representations around *chst4* in *Xenopus*

1. In *X. tropicalis* v10, *chst4* was annotated as *LOC100496630*.
2. In *X. tropicalis* v9.1, *chst4* was annotated as *chst5*.
3. In *X. laevis* v9.2, *chst4.L* was annotated as *chst5.L*.
4. In *X. laevis* v9.2, *chst4.S* was annotated as *chst5.S*.

## Figure S2 Genome browser representations around *chst5.1* in *Xenopus*

1. In *X. tropicalis* v10, *chst5.1* was annotated as *chst6*.
2. In *X. laevis* v9.2, *chst5.1.L* was annotated as *chst6.L*.
3. In *X. laevis* v9.2, *chst5.1.S* was annotated as *chst6.S*.

## Figure S3 Genome browser representations around *chst5.2* in *Xenopus*

1. In *X. tropicalis* v10, *chst5.2* was not annotated.
2. In *X. tropicalis* v9.1, *chst5.2* was annotated as *LOC100485834*.
3. In *X. laevis* v9.2, *chst5.2.L* was annotated as *LOC108714029*.
4. In *X. laevis* v9.2, *chst5.2.S* was annotated as *LOC108715076*.

## Figure S4 Genome browser representations around *chst4/5-like* in *Xenopus*

1. In *X. tropicalis* v10, *chst4/5-like* was annotated as *chst4*.
2. In *X. laevis* v9.2, *chst4/5-like.S* was annotated as *chst4.S*. *chst4/5-like.L* was not found in the genome.

## Figure S5 Genome browser representations around *chst16* in *Xenopus*

1. In *X. tropicalis* v10, *chst16* was annotated as *LOC100485856.*
2. In *X. laevis* v9.2, *chst16.L* was annotated as *LOC108710801*. Genes in the vicinity of *chst16.L* (*tmem263.L* and *cry1.L*) showed strong expression in otic vesicles. An ohnolog of *cry1.L*, *cry2.L*, is located near *chst1.L* and is also expressed strongly in otic vesicles. Forming a genomic regulatory block with these genes, *chst16* may be expressed in otic vesicles.
3. In *X. laevis* v9.2, *chst16.S* was not annotated in the genome.

## Figure S6 Time-course transcriptomic analysis of *chst1, 3, 16*

Expression levels (TPM, transcripts per million) of *chst1*, *chst3*, and *chst16* were plotted against time after fertilization. Unlike *chst1* and *chst3*, *chst16* is not significantly expressed in early *Xenopus* embryos.

## Figure S7 Validation of translational block by *chst1* and *chst5.1* MOs

1. Sequence alignments of *chst1* wild-type sequence (WT) and *chst1* mutated sequence (mt) with *chst1* MO1 and *chst1* MO2 sequence. The start codon (ATG) is underlined.
2. Western blot of FLAG-tagged Chst1 protein produced by an *in vitro* translation system (Promega L2081). WT or mt constructs of *chst1* were mixed with control or *chst1* MOs to achieve an MO concentration of 100 μM. The result indicates that *chst1* MOs inhibits translation of Chst1 in a sequence-specific manner.
3. HSKS immunostaining of *chst1* morphants demonstrated that both *chst1* MO1 and *chst1* MO2 inhibited HSKS synthesis in otic vesicles (open arrowheads), but not in the notochord. Numbers of embryos with observed phenotypes are indicated.

(D,E) Translational block by *chst5.1* MO1 was validated as well as that by *chst1* MOs (A, B).

1. A bent axis phenotype was observed in *chst5.1* morphants. Numbers of embryos with observed phenotypes are indicated. All embryos injected with *chst5.1* MO2 died during gastrulation, even when small amount (0.25 pmol) was injected, possibly due to its high cytotoxicity.

## Figure S8 Validation of splicing and translational block by *chst3* MOs

1. Schematic representation of PCR primers and *chst3* MO on the exon-intron structure of *chst3* gene. Blue and orange boxes indicate UTR and protein coding sequences.
2. RT-PCR of *chst3* mRNA with ex1-F and ex2-R primers using control morphants, *chst3* morphants, and an uninjected control embryo (U). Equivalent sizes and amounts of PCR products were detected in all embryos, indicating that *chst3* MO does not inhibit transcription and splicing of the first intron of *chst3*.
3. RT-PCR of *chst3* mRNA with ex1-F and ex3-R primers using control morphants, *chst3* morphants, and an uninjected control embryo (U). 1-kb larger PCR products were detected from *chst3* morphants than those from control samples, indicating that *chst3* MO inhibits splicing of the second intron of *chst3*, leading to malfunction of Chst3.

(D,E) Translational block by *chst3* MO2 and *chst3* MO3 was validated as well as that by *chst1* MOs (Fig. S7A, B).

1. HSKS immunostaining of *chst3* morphants demonstrated that both *chst3* MO1 and *chst3* MO3 eliminated HSKS in the notochord (open arrowheads), but not in otic vesicles by the later stage (st42) although embryos injected with *chst3* MO3 retained moderate HSKS (grey arrowhead) at the earlier stage (st35/36). Embryos injected with *chst3* MO2 died during gastrulation, even when a small amount (0.5 pmol) was injected, possibly due to its high cytotoxicity. Embryos injected with *chst3* MO3 at 1 pmol/embryo also showed severe developmental defects during organogenesis, whereas those at 0.5 pmol/embryo appeared to develop normally. The smaller injection amount of *chst3* MO3 might have resulted in incomplete inhibition of HSKS synthesis. Numbers of embryos with observed phenotypes are indicated.

## Figure S9 Genome editing of the *chst1* locus reproduced loss of HSKS phenotypes in *Xenopus* embryos.

(A) The protein coding sequence of *chst1* is represented together with its translated amino acid sequence. Forward (5’) and reverse (3’) primer sequences, which were used to amplify target DNA by PCR in the following assays, are highlighted with light blue. Target sequences of sgRNAs (*chst1*-sgRNA1-4) are highlighted with yellow and their corresponding PAM sequences are highlighted with green. Magenta and blue letters of the translated amino acid sequence indicate 5’-PSB and 3’-PB, respectively (see Figure S14). (B) An *in vitro* cleavage assay validated enzymatic activity of the Cas9-sgRNA complex to digest target DNA. Digested DNA fragments were detected as expected. C, control (w/o sgRNA); 1, *chst1*-sgRNA1; 2, *chst1*-sgRNA2; 1+2, *chst1*-sgRNA1+2; 3, *chst1*-sgRNA3; 4, *chst1*-sgRNA4; and M, 100 bp ladder. (C-E) T7E1 assays validated the genome editing efficiency in embryos injected with Cas9 protein preincubated with *chst1-*sgRNAs. Five embryos were examined separately in each experiment. PCR fragments digested by T7E1 enzyme at positions of sgRNA2 and sgRNA4 were detected in *chst1* CRISPRants, but not in uninjected embryos. This indicates that these CRISPRants harbor mutations in the coding sequence of *chst1*. F, F-primer; R, R-primer; 2, sgRNA2; and 4, sgRNA4. (F) HSKS staining of *chst1* CRISPRants demonstrated that loss of function of *chst1* by mutagenesis led to loss of HSKS in otic vesicles, in consistent with *chst1* morphants (Fig. S7C). Due to the mosaicism of mutated cells in CRISPRants, some *chst1* CRISPRants retained HSKS in otic vesicles to some extent. (G) Otic vesicle sizes in CRISPRants were quantified as well as in Fig. 3C. phenotypes. Each value (length of left vesicle, length of right vesicle, width of left vesicle, and width of right vesicle) was statistically analyzed with one-way ANOVA (P < 7.4E^-8^, 1.15E^-5^, 4.56E^-4^, and 9.77E^-5^, respectively), followed by Tukey’s honestly significant difference test with 95% confidence level (indicated with a and b). This result further demonstrates that Chst1 regulates otic vesicle morphogenesis via HSKS synthesis in *Xenopus* embryos.

## Figure S10 Genome editing of the *chst3* locus reproduced loss of HSKS phenotypes in *Xenopus* embryos.

(A) The protein coding sequence of *chst3* is represented together with its translated amino acid sequence, as in Fig. S9. An intron inserted position is designated by an arrow (see Fig. S7). (B) An *in vitro* cleavage assay validated enzymatic activity of the Cas9-sgRNA complex to digest target DNA. Digested DNA fragments were detected as expected. C, control (w/o sgRNA); 1, *chst3*-sgRNA1; 2, *chst3*-sgRNA2; 1+2, *chst3*-sgRNA1+2; 3, *chst3*-sgRNA3; 4, *chst3*-sgRNA4; and M, 100 bp ladder. (C-E) T7E1 assays validated the genome editing efficiency in embryos injected with Cas9 protein preincubated with *chst3*-sgRNAs. Five embryos were examined separately in each experiment. As a result, PCR fragments were significantly digested by T7E1 enzyme only at the position of *chst3*-sgRNA3. This indicates that CRISPRants with *chst3*-sgRNA3 harbor mutations in the coding sequence of *chst3*. F, F-primer; R, R-primer; and 3, sgRNA3. (F) HSKS staining of *chst3* CRISPRants revealed that notochordal HSKS was eliminated from CRISPRants with *chst3*-sgRNA3, in consistent with *chst3* morphants (Fig. S8F).

## Figure S11 Genome editing further confirmed that *chst5.1* functions in HSKS synthesis in *Xenopus* embryos.

(A) The protein coding sequence of *chst5.1* is represented together with its translated amino acid sequence, as in Fig. S8. (B) An *in vitro* cleavage assay validated enzymatic activity of the Cas9-sgRNA complex to digest target DNA. Both *chst5.1*-sgRNA1 and *chst5.1*-sgRNA2 showed sufficient cleavage activity. (C-E) A T7E1 assay validated the genome editing efficiency in embryos injected with Cas9 protein preincubated with *chst5.1*-sgRNA1 (C), *chst5.1-*sgRNA2 (D), or both (E). Five embryos were examined separately in each experiment. PCR fragments digested by T7E1 enzyme at each sgRNA position were detected in CRISPRants, but not in uninjected embryos, indicating that these CRISPRants harbor mutations in the coding sequence of *chst5.1*. F, F-primer; R, R-primer; 1, sgRNA1; and 2, sgRNA2. (E) HSKS staining of *chst5.1* CRISPRants validated that *chst5.1* is required for HSKS synthesis in the notochord and otic vesicles. CRISPRants with *chst5.1*-sgRNA2 showed relatively weaker phenotypes than those with *chst5.1*-sgRNA1, although their enzymatic activity and genome editing efficiencies are equivalent (B-E). A part of CRISPRants with *chst5.1*-sgRNA1+2 exhibited almost complete loss of HSKS in otic vesicles, but others did not. These variable phenotypes suggest that HSKS can be synthesized by small number of normal cells/enzymes in otic vesicles. Open arrowheads indicate lost or reduced enrichment of HSKS.

## Figure S12 ORTHOSCOPE reported putative Chst genes in deuterostomes

An NJ tree of putative Chst genes in deuterostomes is reported using ORTHOSCOPE. *Drosophila* genes were used as the outgroup. The color code for taxonomy of gene names is as follows: black, protostomes; purple, echinoderms; green, hemichordates; orange, cephalochordates (amphioxus); magenta, tunicates; blue, vertebrates.

## Figure S13 ML tree of putative deuterostome Chst genes

An ML tree was constructed using the same set of sequences and color codes for gene names as in Fig. S6. *Drosophila* genes were used as the outgroup. Genes with notochordal expression are highlighted in light blue for *Xenopus*, *Branchiostoma*, and *Ciona* genes.

## Figure S14 Adenosine 3’-phosphate 5’-phosphosulfate binding motifs are widely conserved among chordate Chst proteins

Sequence alignments of chordate Chst proteins indicate conservation of 5’-phosphosulfate binding motifs (5’-PSB) and 3’-phosphate binding motifs (3’-PB). Core motifs, RS/TGSSF in 5’-PSB and RDPR in 3’-PB, are widely conserved among chordate Chst proteins, whereas some residues of 5’-PSB and 3’-PB have changed specifically to each ohnolog, which may have caused differentiation of substrates. Xtr-Chst5.2 sequence was obtained from the record XP_012817703, which was now removed as a result of standard genome annotation processing. Mutations in the core motif of 5’-PSB and longer branch length in the phylogenetic tree (see Figs. S11 and S12) imply that *chst5.2* is a dying gene. Hsa, *Homo sapiens*; Xtr, *Xenopus tropicalis*; Dre, *Danio rerio*; Cin, *Ciona intestinalis*; Bfl, *Branchiostoma floridae*.
